# Assessment of the Therapeutic Potential of Enhancer of Zeste Homolog 2 Inhibition in a Murine Model of Bronchiolitis Obliterans Syndrome

**DOI:** 10.3389/ti.2024.13227

**Published:** 2024-10-25

**Authors:** Kyoto Matsudo, Shinkichi Takamori, Tomoyoshi Takenaka, Mototsugu Shimokawa, Asato Hashinokuchi, Taichi Nagano, Fumihiko Kinoshita, Takaki Akamine, Mikihiro Kohno, Gouji Toyokawa, Tomoharu Yoshizumi

**Affiliations:** ^1^ Department of Surgery and Science, Graduate School of Medical Sciences, Kyushu University, Fukuoka, Japan; ^2^ Department of Thoracic and Breast Surgery, Oita University Faculty of Medicine, Oita, Japan; ^3^ Department of Biostatistics, Yamaguchi University Graduate School of Medicine, Yamaguchi, Japan; ^4^ Department of Thoracic Surgery, Clinical Research Institute, National Hospital Organization, Kyushu Medical Center, Fukuoka, Japan

**Keywords:** bronchiolitis obliterans syndrome, chronic lung allograft dysfunction, 3-deazaneplanocin A, enhancer of zeste homolog 2, lung transplantation

## Abstract

Bronchiolitis obliterans syndrome (BOS) is a chronic complication following lung transplantation that limits the long-term survival. Although the enhancer of zeste homolog 2 (EZH2) is involved in post-transplantation rejection, its involvement in BOS pathogenesis remains unclear. We aimed to investigate the therapeutic potential of EZH2 inhibition in BOS. 3-deazaneplanocin A (DZNep) was administered intraperitoneally to heterotopic tracheal transplant recipient model mice. Tracheal allografts were obtained on days 7, 14, 21, and 28 after transplantation. The obstruction ratios of the DZNep and control groups on days 7, 14, 21, and 28 were 15.1% ± 0.8% vs. 20.4% ± 3.6% (*p* = 0.996), 16.9% ± 2.1% vs. 67.7% ± 11.5% (*p* < 0.001), 47.8% ± 7.8% vs. 92.2% ± 5.4% (*p* < 0.001), and 60.0% ± 9.6% vs. 95.0% ± 2.3% (*p* < 0.001), respectively. The levels of interleukin (IL)-6 and interferon-γ on day 7 and those of IL-2, tumor necrosis factor, and IL-17A on days 14, 21, and 28 were significantly reduced following DZNep treatment. DZNep significantly decreased the number of infiltrating T-cells on day 14. In conclusion, DZNep-mediated EZH2 inhibition suppressed the inflammatory reactions driven by pro-inflammatory cytokines and T cell infiltration, thereby alleviating BOS symptoms.

## Graphical Abstract

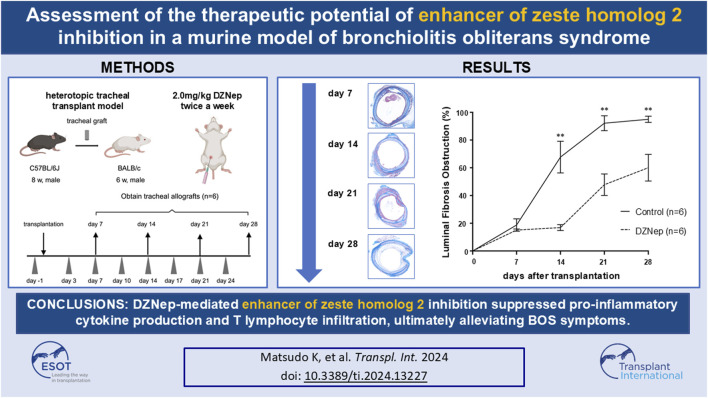

## Introduction

As a last resort for severe respiratory conditions, lung transplantation is the treatment of choice for patients with progressive lung disease and irreversible pulmonary failure when no other effective treatments are available and the patient’s life is at risk. However, long-term survival rates remain low owing to chronic lung allograft dysfunction (CLAD) [1]. Chronic lung allograft dysfunction has four subtypes: bronchiolitis obliterans syndrome (BOS), restrictive allograft syndrome, mixed CLAD, and undefined CLAD [2].

Chronic lung allograft dysfunction is defined as a substantial and persistent decline (≥20%) in forced expiratory volume in 1 s (FEV_1_) relative to the reference FEV_1_ [2]. FEV_1_ is the average of the two best postoperative FEV_1_ readings, taken at least 3 weeks apart [3], after excluding other pulmonary and extrapulmonary causes for FEV_1_ decline. The most common manifestation of CLAD is airflow limitation caused by BOS. Previously, the diagnostic criteria for CLAD were applied to BOS. Now, BOS is defined by a decline of FEV1 (≥20%) from the previous baseline, a ratio of FEV1 to forced vital capacity <0.7, and no opacities on chest imaging [4]. According to a recent report, the median time from BOS onset to death or retransplantation was 500 days [5]. Although BOS was first introduced in 1993 [3] and clinically defined in 2003 [6], the therapeutic options for this condition remain limited and lack a clearly established protocol. A consensus guideline for the diagnosis and treatment of BOS published in 2014 evaluated the existing literature and used the Grading of Recommendations, Assessment, Development, and Evaluation system to demonstrate that most BOS therapies were inadequate [7]. Thus, developing more effective treatment options for patients with BOS is crucial.

The enhancer of zeste homolog 2 (EZH2), which methylates histone H3 on lysine 27 (H3K27me3), regulates cellular differentiation via histone methylation [8]. Different types of EZH2 inhibitors have been developed [9], and some are already used in clinical practice to treat malignancies, including B-cell lymphoma [10]. The importance of EZH2-targeted treatment in managing acute and chronic rejection post-transplantation is increasingly recognized. For instance, EZH2 inhibition has been shown to suppress acute renal allograft rejection in a rat model [11]. Additionally, Zaiken et al. reported that EZH2 inhibition improved pulmonary function in a chronic graft-versus-host disease mouse model with bronchiolitis obliterans [12]. In these studies, inhibiting or deleting EZH2 was shown to suppress the differentiation and functions of immune cells, especially T cells. Given the critical roles of inflammatory reactions in the acute phase and immune rejection in the chronic phase in triggering BOS formation [13], we hypothesized that inhibiting EZH2 could comprehensively control these reactions and suppress BOS.

3-deazaneplanocin A (DZNep) is an inhibitor of S-adenosylhomocysteine hydrolase that inhibits H3K27 methylation and the activity of EZH2 [10]. Although DZNep has not yet been applied clinically in humans, it has garnered attention for its various potential benefits [14]. In this study, we aimed to assess the effects of DZNep-mediated EZH2 inhibition on BOS using a murine heterotopic tracheal transplant (HTT) model.

## Materials and Methods

### Induction of the Murine BOS Model

**FIGURE 1:**
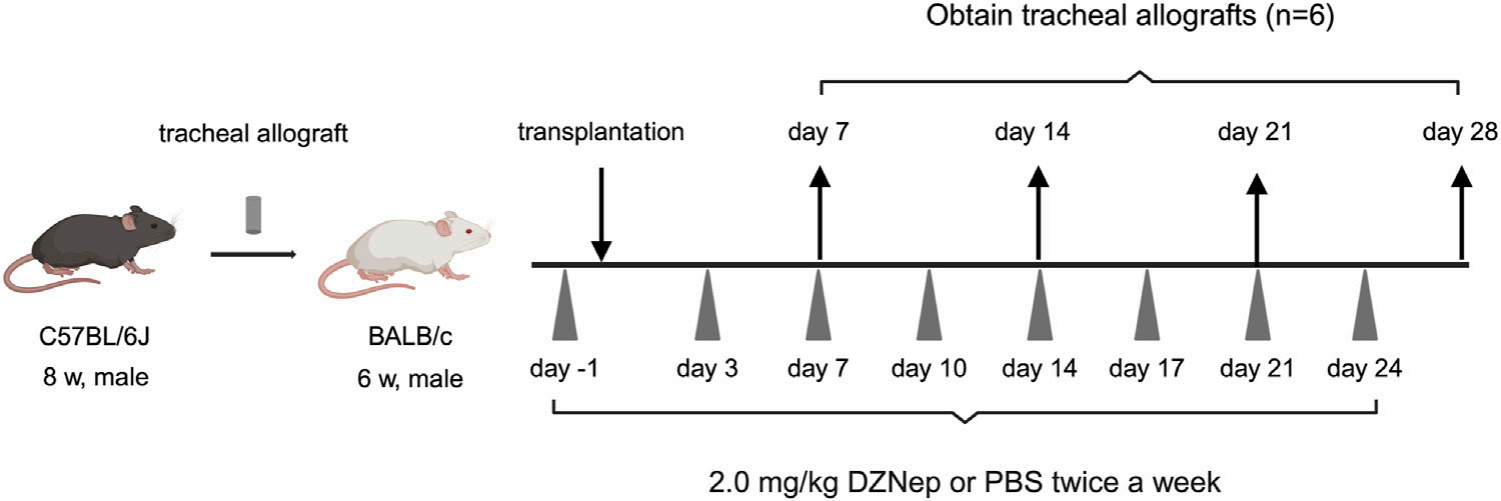
A schematic overview of the murine heterotopic tracheal transplant model experiments. Briefly, recipient mice were intraperitoneally injected with either 2.0 mg/kg DZNep suspended in 400 μL of PBS or 400 μL of PBS alone on days −1, 3, 7, 10, 14, 17, 21, and 24. Tracheal allografts were harvested on the indicated days after transplantation. DZNep, 3-deazaneplanocin A; PBS, phosphate-buffered saline.

Male 8-week-old C57BL/6 J and 6-week-old BALB/c mice were purchased from Kyudo Ltd. Animals were housed in a specific pathogen-free facility at Kyushu University, Japan. All mice received humane care in compliance with the Principles of Laboratory Animal Care formulated by the National Society for Medical Research and the “Guide for the Care and Use of Laboratory Animals” prepared by the Institute of Laboratory Animal Resources and published by the National Institutes of Health. The Institutional Review Board approved the animal experiments (No. A22-291-0). A well-established HTT model was used in this study (Figure 1) [15–17]. Briefly, donor mice were anesthetized with isoflurane, and euthanized by cervical dislocation. After being placed in a supine position, a midline cervical incision was made to expose the entire trachea. Tracheal allografts were taken from the first tracheal ring to the carina, such that each tracheal segment was >8 mm. Tracheal allografts were suspended in phosphate-buffered saline (PBS) and stored on ice until implantation. The recipient mice were anesthetized using isoflurane. Donor tracheal allografts were transplanted into a subcutaneous pouch created on the dorsal site of the recipient mice. The skin was closed with sutures.

DZNep was purchased from Nacalai Tesque (Kyoto, Japan). To evaluate the role of EZH2 inhibition in BOS, recipient mice were intraperitoneally injected with 2.0 mg/kg DZNep suspended in 400 μL of PBS 1 day before transplantation and subsequently on days 3, 7, 10, 14, 17, 21, and 24 post-transplantation (twice a week). The dosing regimen for DZNep was established based on previous studies [18, 19], which confirmed the absence of serious adverse events in the test administrations. Control mice received the same frequency and dose of PBS intraperitoneally as the DZNep mice to ensure comparable conditions.

Grafts were obtained on days 7, 14, 21, and 28 post-transplantation (n = 6 per group). All the grafts were cut in half. Half of the tracheae were fixed in 4% paraformaldehyde for 24 h at room temperature for histopathological assessment, whereas the other half were stored as frozen specimens and used for cytokine expression by flow cytometry.

### Histopathologic Evaluation

Formalin-fixed tissues were paraffin-embedded and cut into 5-μm sections. The slides were stained with hematoxylin and eosin and Masson’s trichrome. The extent of luminal obstruction in the trachea was calculated using Masson’s trichrome staining, according to the following formula: (area obstructed by fibrotic tissue)/(area within the cartilage) × 100%. The obstruction ratio was assessed using ImageJ 1.50 software (National Institutes of Health, Bethesda, MD, United States).

### Quantification of Cytokine Production

Frozen tracheal allografts were homogenized in radioimmunoprecipitation assay buffer containing sodium dodecyl sulfate (Nacalai Tesque, Kyoto, Japan). The concentrations of interleukin (IL)-2, IL-4, IL-6, interferon (IFN)-γ, tumor necrosis factor (TNF), IL-17A, and IL-10 in the allografts were simultaneously measured using the BD Cytometric Bead Array Mouse Th1/Th2/Th17 Cytokine Kit (560485; BD Biosciences, San Diego, CA, United States) on a FACSuite flow cytometer (BD Biosciences). The assays were performed and analyzed by a single operator using CBA Analysis Software 1.1.14.

### Immunohistochemical Staining

Immunohistochemical staining for EZH2, CD8, and CD4 was performed on 4-μm formalin-fixed and paraffin-embedded tissues according to the manufacturer’s instructions. The sections were deparaffinized, blocked with 10% normal goat serum, and incubated with the following primary polyclonal antibodies at 4°С overnight: rabbit monoclonal anti-EZH2 antibody (1:100 dilution, Abcam, Cambridge, United Kingdom), rabbit monoclonal anti-CD8 (1:2000 dilution, Abcam), and rabbit monoclonal anti-CD4 (1:1,000 dilution, Abcam). The immune complexes were detected using the Dako EnVision Detection System (Dako, Glostrup, Denmark). Finally, the sections were treated with 3,3-diaminobenzidine, counterstained with hematoxylin, and mounted. EZH2 protein levels were measured by counting the number of EZH2-positive cells in each high-power field (×1,000) and averaging over at least five fields per graft, with six mice per group. The extent of CD8^+^ and CD4^+^ T-cell infiltration was evaluated by counting the number of CD8^+^ and CD4^+^ cells in each high-power field (×1,000) and averaging over at least five fields per graft and six mice per group.

### Statistical Analysis

Data were expressed as mean ± standard error of the mean. One-way analysis of variance was used to compare multiple groups, and Student’s t-test was used to compare two groups. Differences were considered statistically significant if the *p*-value was <0.05. All analyses were conducted using the JMP^®^ 16.0 software (SAS Institute, Cary, NC, United States).

## Results

### Luminal Fibrous Occlusion in Untreated Allografts After HTT

**FIGURE 2:**
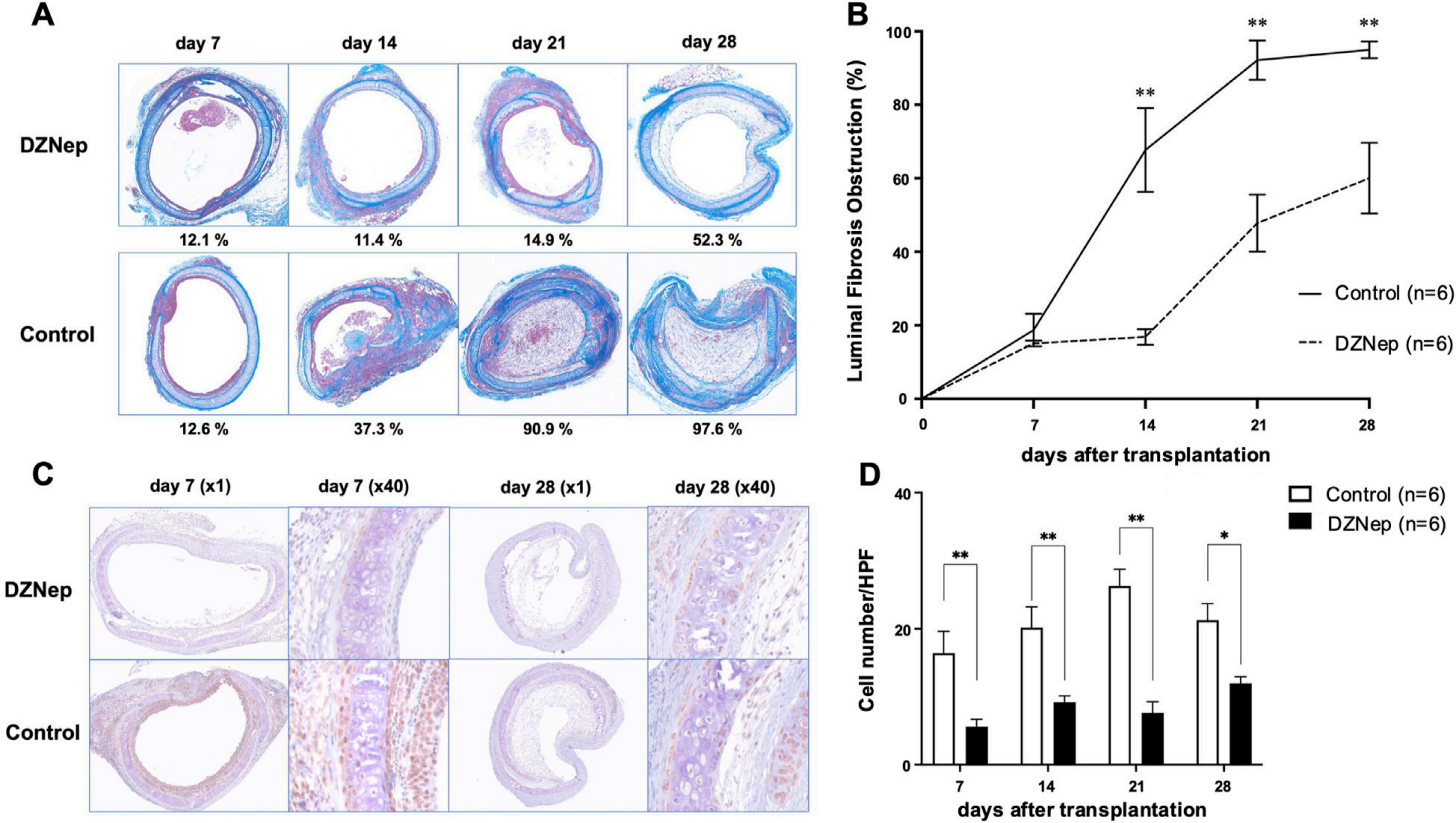
DZNep treatment reduces the extent of tracheal lumen occlusion following transplantation. **(A)** Representative Masson’s trichrome staining images of the pathological features of heterotopically transplanted tracheal allografts in the DZNep and control groups on days 7, 14, 21, and 28 after transplantation (original magnification, ×100). **(B)** Analysis of luminal fibrous occlusion in the DZNep and control groups (n = 6 per group) at the indicated timepoints. **(C)** Representative images of EZH2 staining in heterotopically transplanted tracheal allografts from the DZNep and control groups on days 7 and 28. **(D)** The number of EZH2-positive cells per high-powered field (×1,000), averaged across at least five fields and six mice (per group). Data represent the mean ± standard error of the mean. DZNep, 3-deazaneplanocin A; EZH2, enhancer of zeste homolog 2. *p < 0.05; **p < 0.01.

Initially, we observed the extent of luminal fibrous occlusion in HTT allografts (Figure 2A). Infiltration of inflammatory cells into the epithelial layer was observed 7 days after transplantation. By day 14, inflammatory cells had reached the tracheal lumen, leading to fibrosis. On day 21, the lumen was almost completely occluded by fibrous connective tissue and extensive inflammatory cell infiltration was observed. The tracheal lumen was occluded on day 28. The mean obstruction ratios on days 7, 14, 21, and 28 were 20.40% ± 3.62%, 67.68% ± 11.46%, 92.15% ± 5.38%, and 94.97% ± 2.28%, respectively (Figure 2B).

### DZNep Treatment Reduces EZH2 Protein Levels and Alleviates Tracheal Luminal Fibrous Occlusion

To investigate whether EZH2 is implicated in BOS pathogenesis, we administered DZNep to recipient mice (Figure 2A). The obstruction ratios of the DZNep group and the control group on days 7, 14, 21, and 28 were 15.1% ± 0.8% vs. 20.4% ± 3.6% (*p* = 0.996), 16.9% ± 2.1% vs. 67.7% ± 11.5% (*p* < 0.001), 47.8% ± 7.8% vs. 92.2% ± 5.4% (*p* < 0.001), and 60.0% ± 9.6% vs. 95.0% ± 2.3% (*p* < 0.001), respectively (Figure 2B). Thus, DZNep significantly reduced the obstruction ratio of the trachea transplanted into HTT model mice.

The protein levels of EZH2 were examined using immunohistochemical staining to confirm the reduced expression of EZH2 in the transplanted trachea following DZNep administration (Figure 2C). EZH2 protein levels were significantly lower in the DZNep treatment group than in the control group on days 7 (5.6 ± 1.1/HPF vs. 16.4 ± 3.2/HPF; *p* = 0.004), 14 (9.2 ± 0.9/HPF vs. 20.2 ± 3.1/HPF; *p* = 0.004), 21 (7.6 ± 1.6/HPF vs. 26.3 ± 2.5/HPF; *p* < 0.001), and 28 (12.0 ± 1.0/HPF vs. 21.2 ± 2.5/HPF; *p* = 0.017) (Figure 2D).

### DZNep Treatment Alters Cytokine Levels in Tracheal Allografts

**FIGURE 3:**
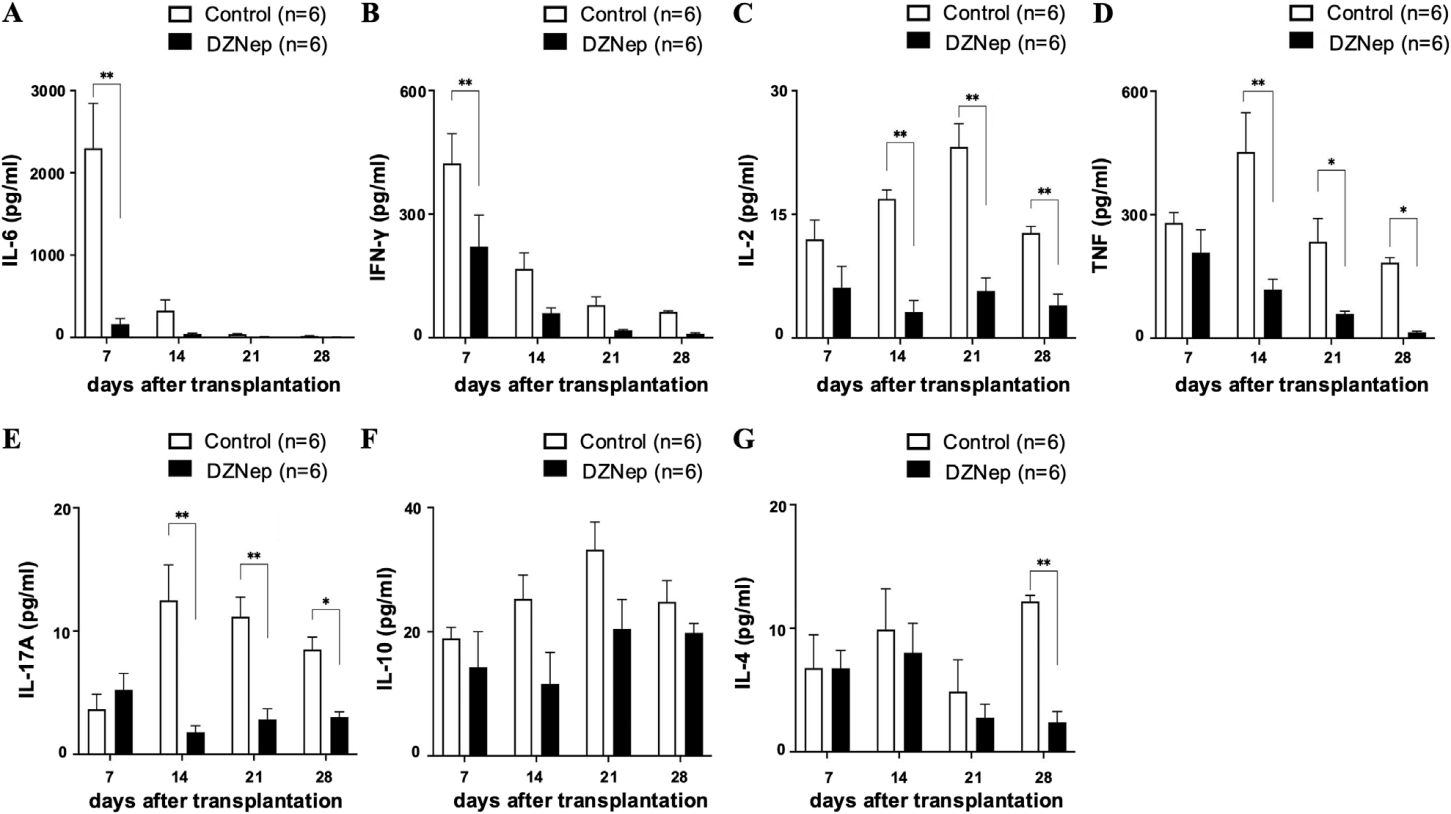
DZNep treatment modifies cytokine levels in tracheal allografts. Analysis of IL-6 **(A)**, IFN-γ **(B)**, IL-2 **(C)**, TNF **(D)**, IL-17A **(E)**, IL-10 **(F)**, and IL-4 **(G)** concentrations in the DZNep and the control groups after heterotopic tracheal transplantation (n = 6 per group). Data represent the mean ± standard error of the mean. DZNep, 3-deazaneplanocin A. IFN-γ, interferon-gamma; IL, interleukin; TNF, tumor necrosis factor. *p < 0.05; **p < 0.01.

Since inflammatory reactions in the acute phase and immune rejection in the chronic phase are considered key triggers of BOS formation, we measured cytokine levels in frozen tracheal allografts on days 7, 14, 21, and 28 post-transplantation. The levels of IL-6 and IFN-γ (two cytokines which exert pleiotropic effects on inflammation and immune responses [20, 21]) were significantly lower in the DZNep group than in the control group on day 7 (IL-6:160.0 ± 67.3 pg/mL vs. 2298.1 ± 546.4 pg/mL; *p* < 0.001, IFN-γ: 221.0 ± 76.6 pg/mL vs. 422.5 ± 72.7 pg/mL; *p* = 0.005) (Figures 3A, B). Meanwhile, the levels of IL-2 (a proinflammatory cytokine which is produced by T helper cell 1 (Th1) lymphocytes and is a potent activator of T cells and natural killer cells [22]) were significantly lower in the DZNep group than in the control group on days 14 (3.2 ± 1.4 pg/mL vs. 16.9 ± 1.1 pg/mL; *p* < 0.001), 21 (5.7 ± 1.6 pg/mL vs. 23.2 ± 2.8 pg/mL; *p* < 0.001), and 28 (4.0 ± 1.3 pg/mL vs. 12.8 ± 0.8 pg/mL; *p* = 0.008) (Figure 3C). The levels of TNF (a pro-inflammatory cytokine that promotes the activation of Th1 lymphocytes, neutrophils, and macrophages [22, 23]) were also significantly reduced in the DZNep group vs. the control group on days 14 (118.2 ± 25.2 pg/mL vs. 452.2 ± 95.6 pg/mL; *p* < 0.001), 21 (59.3 ± 6.6 pg/mL vs. 234.5 ± 56.4 pg/mL; *p* = 0.040), and 28 (14.0 ± 3.0 pg/mL vs. 183.4 ± 12.7 pg/mL; *p* = 0.050) (Figure 3D). The levels of IL-17A (a pro-inflammatory cytokine produced by T helper 17 (Th17) cells that promotes the migration of inflammatory cells [21]) were also lower in the DZNep group than in the control group on days 14 (1.8 ± 0.5 pg/mL vs. 12.5 ± 2.9 pg/mL; *p* < 0.001), 21 (2.8 ± 0.9 pg/mL vs. 11.2 ± 1.6 pg/mL; *p* < 0.001), and 28 (3.6 ± 0.2 pg/mL vs. 8.5 ± 1.0 pg/mL; *p* = 0.039) (Figure 3E).

We found no significant difference in the levels of IL-10 (an anti-inflammatory cytokine [24]) between the DZNep and control groups at any of the timepoints (Figure 3F). However, the production of IL-4 (a cytokine with an important role in T helper 2 (Th2) cell differentiation [21]) was significantly suppressed on day 28 in the DZNep group vs. the control group (2.4 ± 0.9 pg/mL vs. 12.2 ± 0.5 pg/mL; *p* = 0.008) (Figure 3G).

### DZNep Reduces T Lymphocyte Infiltration Into the Allograft

**FIGURE 4:**
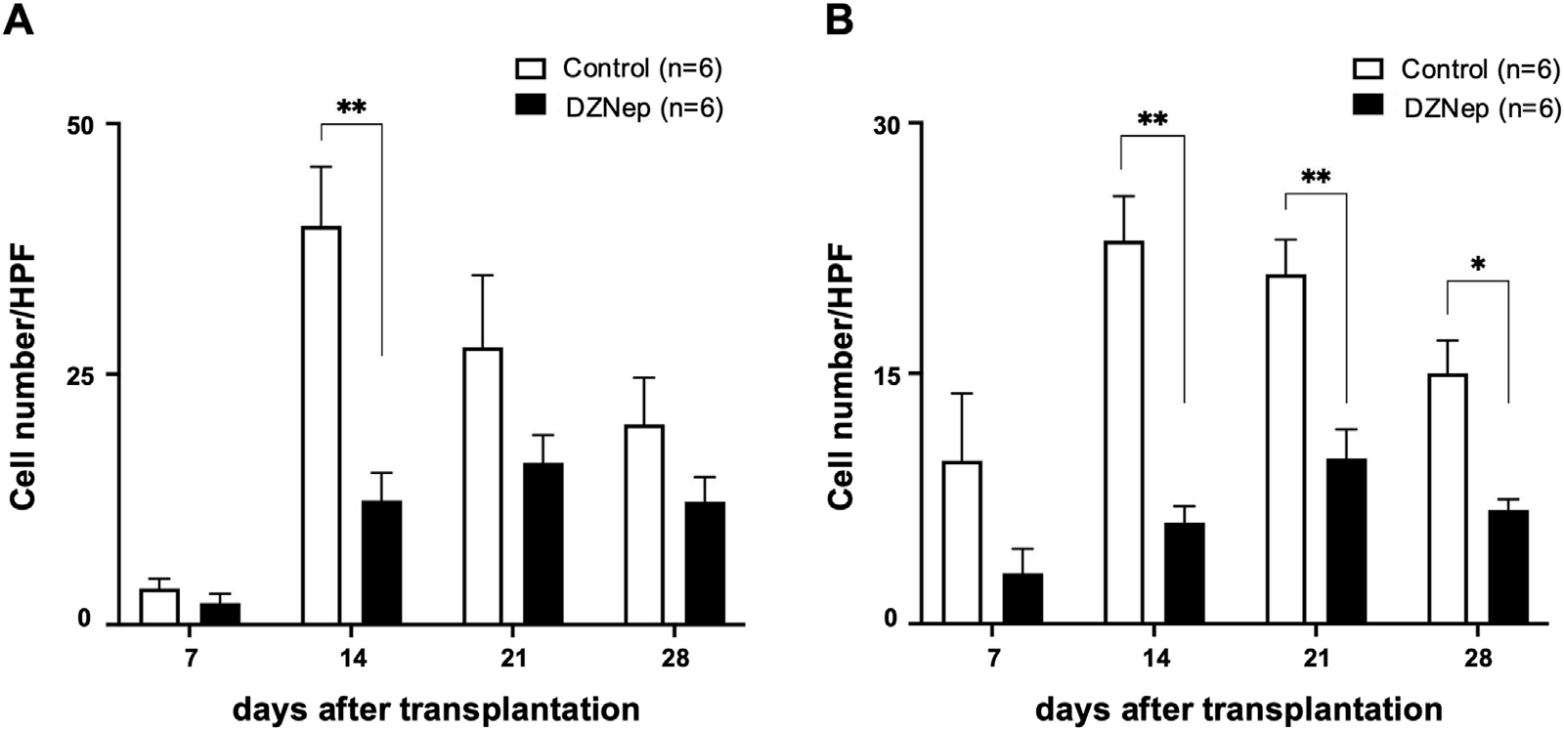
Effect of EZH2 inhibition on T cell infiltration. The number of CD8^+^ **(A)**, and CD4^+^ **(B)** T cells per high-powered field (×1,000) averaged across at least five fields and six mice (per group). Data represent the mean ± standard error of the mean. EZH2, enhancer of zeste homolog 2. *p < 0.05; **p < 0.01.

Immunohistochemical staining for CD8 and CD4 was performed on the tracheal allografts to determine changes in the distribution of T lymphocytes after transplantation. The numbers of CD8^+^ and CD4^+^ T lymphocytes infiltrating the allografts were significantly lower in the DZNep group than in the control group on day 14 (CD8: 12.4 ± 2.8/HPF vs. 39.8 ± 5.9/HPF; *p* < 0.001, CD4: 6.1 ± 1.0/HPF vs. 22.9 ± 2.7/HPF; *p* < 0.001) (Figures 4A, B). Although no significant difference was observed in the number of infiltrating CD8^+^ T lymphocytes between the groups at the later timepoints, the number of CD4^+^ T cells was significantly lower on days 21 (9.9 ± 1.7/HPF vs. 20.9 ± 2.1/HPF; *p* = 0.004) and 28 (6.8 ± 0.6/HPF vs. 15.0 ± 2.0/HPF; *p* = 0.045) owing to DZNep treatment (Figure 4B).

## Discussion

Lung transplantation is the most effective treatment for patients with severe or terminal lung diseases. However, despite clinical advances, the outcomes of lung transplantation remain worse than those of other solid organ transplantations. The overall survival of lung transplantation patients at 5 years is only approximately 50%–70% [25, 26]. Obliterative bronchiolitis (OB) is a pathological condition characterized by airflow limitation due to scarring or the filling of the airway lumen with a collagen matrix. BOS is a clinical syndrome with the pathogenesis of OB [27]. For patients with BOS who died or underwent retransplantation, the median time from BOS onset to death or retransplantation was 500 days [5]. This duration varies depending on the time of BOS onset [28, 29]. The pathogenesis of BOS has three stages. In the first phase, immune and/or non-immune factors (such as acute rejection and lymphocytic bronchiolitis) damage the airway epithelium. Subsequently, infiltrating immune cells are stimulated to produce various cytokines and chemokines, initiating an inflammatory cascade. Finally, persistent inflammation drives bronchiolar tissue remodeling, resulting in fibrosis and airway lumen occlusion [13, 30]. Controlling inflammatory reactions during the acute phase is crucial for reducing the pathogenesis of BOS.

In this study, we used an HTT model (which was first developed for the investigation of OB in 1993 [16]) to investigate the effects of EZH2 on BOS. In this model, tracheal allografts follow a process similar to that of OB, with lumen closure. The process initially involves the loss of epithelial cells, which is followed by the induction of inflammatory cells in the grafts [31, 32]. Excessive fibroblast/myofibroblast proliferation results in total occlusion of the intratracheal region [17]. Since the HTT model is suitable for investigating immunological changes, epithelial damage/regeneration, and fibrosis [33], as noted above, we used this model to observe whether EZH2 inhibition could prevent inflammatory cell infiltration and fibrotic obstruction.

**FIGURE 5:**
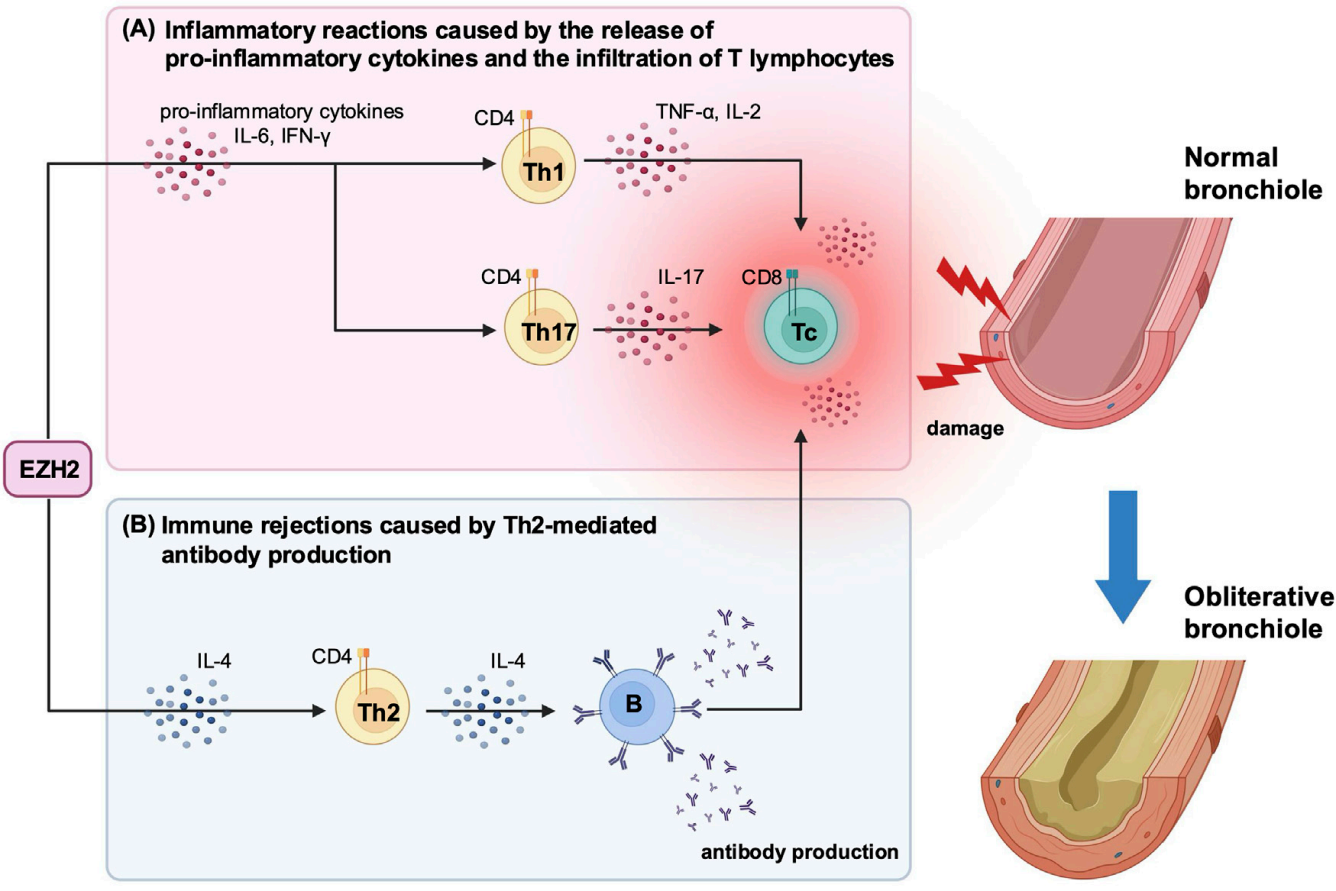
A schematic diagram illustrating the immunomodulatory effects of EZH2-mediated cytokine production following heterotopic trachea transplantation. **(A)** In the early post-transplantation period, EZH2 promotes the production of pro-inflammatory cytokines, including IL-6 and IFN-γ. These cytokines stimulate further release of pro-inflammatory cytokines and the infiltration of T lymphocytes. **(B)** In the late post-transplantation period, EZH2 sustains the production of IL-4, which drives Th2-mediated antibody production. This antibody production induces the recruitment of immune cells, leading to graft dysfunction and tissue damage. These inflammatory reactions and immune rejections are key triggers for BOS development. DZNep-mediated EZH2 inhibition significantly suppresses the production of both pro-inflammatory cytokines and IL-4 post-transplantation, which may reduce the risk of developing BOS and protect the graft from immune-mediated damage. BOS, bronchiolitis obliterans syndrome; DZNep, 3-deazaneplanocin A; EZH2, enhancer of zeste homolog 2, IFN-γ, interferon-gamma; IL, interleukin; Th, T helper cell; TNF, tumor necrosis factor.

EZH2 has important roles in the regulation of various cellular functions, including development and differentiation [8, 34]. One of its functions is to promote the differentiation and infiltration of inflammatory cells by activating the signal transducer and activator of transcription 3 (STAT3) [35]. Zhang et al. reported that inhibiting the STAT3 signaling pathway by blocking EZH2 reduces inflammatory cell infiltration and cytokine release in a cecal ligation and puncture mouse model [36]. Consistent with these findings, our study suggests that EZH2 plays an important role in the pathogenesis of BOS by inducing pro-inflammatory cytokines production and T-lymphocyte infiltration during both the early and late post-transplantation periods (Figure 5). Specifically, we showed that DZNep treatment significantly suppressed the release of IL-6 and IFN-γ on day 7 post-transplantation, and the production of other pro-inflammatory cytokines (including IL-2, TNF, and IL-17A) on days 14, 21, and 28. DZNep administration also reduced the infiltration of CD8^+^ and CD4^+^ T lymphocytes into the allograft, with peak suppression observed at 14 days post-transplantation. We demonstrated that EZH2 inhibition prevented the inflammatory reactions triggered by the release of pro-inflammatory cytokines and T cell infiltration, potentially protecting the allograft in the early post-transplantation period. Although not significant, CD8^+^ T lymphocytes were reduced on days 21 and 28, and pro-inflammatory cytokine levels were significantly low after day 21. These findings may contribute to long-term allograft survival.

Multiple cytokines are implicated in OB development, among which the role of IL-17 in BOS has been reported by many studies [37, 38]. For instance, IL-17 participates in the pathogenesis of OB by regulating macrophage polarization in a murine HTT model [37]. Meanwhile, blocking IL-17A reduces the overall IFN-γ-mediated lymphocyte response and decreases the likelihood of OB development [38]. Furthermore, IFN-γ alone appears to be closely associated with airway inflammation and fibrosis following lung transplantation [39, 40]. Elevated IL-6 concentrations are also correlated with BOS [41]. Thus, the therapeutic effect of EZH2 inhibition likely stems from its role as an epigenetic regular, which leads to increase BOS-associated pro-inflammatory cytokine production (Figure 5).

In this study, IL-4 levels were significantly suppressed in the DZNep group on day 28. Additionally, the number of CD4^+^ T lymphocytes was significantly reduced in the DZNep group after day 14. These results suggest that DZNep treatment may suppress Th2-mediated antibody production during the late post-transplantation period. Similar to these findings, several studies have reported that EZH2 both directly and indirectly regulates antibody production from B cells through Th2 cells [42, 43]. Antibody binding triggers both complement-dependent and complement-independent recruitment of immune cells, which can lead to graft dysfunction and tissue damage post-transplantation [44]. Antibody-mediated rejection (AMR) is also recognized as a predictor of CLAD development, with new therapies aimed at reducing AMR risk currently under investigation [4]. The suppression of antibody production through DZNep-mediated EZH2 inhibition could prevent Th2-mediated immune rejection in the chronic phase and enhance long-term allograft survival, although further validation is needed.

To the best of our knowledge, this is the first study to demonstrate the potential of DZNep-mediated EZH2 inhibition in BOS. DZNep has several advantages over traditional immunosuppressive agents such as cyclosporine A and tacrolimus. First, it exhibits broad-spectrum and potent antiviral activity, including against human cytomegalovirus [45, 46], which causes serious infections in immunocompromised transplant recipients [46]. Second, pharmacological EZH2 inhibition by DZNep is associated with beneficial therapeutic effects in several cancers [9, 47]. Given that the long-term use of immunosuppressive agents after transplantation increases the risk of malignancy, the antitumor effects of DZNep should not be overlooked. Thus, DZNep is a promising therapeutic agent for organ transplantation; nevertheless, its efficacy and safety warrant further investigation in clinical studies.

The murine HTT model used in this study has some shortcomings. Notably, this model lacks blood vessels and an interface with air. Although a single-lung transplant mouse model has been successfully created [48, 49], we used an HTT model in this study due to its superiority in terms of high OB reproducibility. Moreover, another reason for adopting this model was the possibility to observe pathological changes in a short period following a simple and easy procedure [33]. However, the role of EZH2 in BOS pathogenesis requires further validation using an alternative model, such as a murine orthotopic transplant model.

Conclusively, we used a murine HTT model to demonstrate that EZH2 plays an important role in BOS pathogenesis. Our findings demonstrate that DZNep-mediated EZH2 inhibition reduces inflammation by suppressing the release of pro-inflammatory cytokines and T cell infiltration during both early and late post-transplantation periods, ultimately reducing the severity of BOS. Collectively, our preclinical results imply that DZNep holds promise as a therapeutic agent for lung transplantation; however, its efficacy and safety must be further validated through rigorous clinical testing.

## Data Availability

The raw data supporting the conclusions of this article will be made available by the authors, without undue reservation.

## Abbreviations

AMR, Antibody-mediated rejection; BOS, bronchiolitis obliterans syndrome; CLAD, chronic lung allograft dysfunction; DZNep, 3-deazaneplanocin A; EZH2, enhancer of zeste homolog 2; FEV1, expiratory volume in 1 second; HTT, heterotopic tracheal transplant; IFN-γ, interferon-gamma; IL, interleukin; OB, obliterative bronchiolitis; PBS, phosphate buffered saline; STAT3, signal transducer and activator of transcription 3; Th, T helper cell; TNF, tumor necrosis factor.
